# Supplementary material for: Dystroglycan is a scaffold for extracellular axon guidance decisions
Source: eLife. 2019 Feb 13;8:e42143. doi: 10.7554/eLife.42143 (PMC6395066; doi:10.7554/eLife.42143)
Supplement: Supplementary file 1. — E12.5 spinal cords were processed for open book preparations and each well-isolated DiI injection site was assessed as showing either normal anterior turning or anterior-posterior randomization. [file elife-42143-supp1.docx]

**Table 1: Open book raw data for *Dag1* mutants**

| Genotype | Embryo # | Injection sites | Normal turn | AP randomization | % normal | Genotype average ±SEM |
| --- | --- | --- | --- | --- | --- | --- |
| Control | 1 | 6 | 6 | 0 | 100 |  |
|  | 2 | 11 | 11 | 0 | 100 |  |
|  | 3 | 13 | 13 | 0 | 100 |  |
|  | 4 | 9 | 9 | 0 | 100 |  |
|  | 5 | 3 | 3 | 0 | 100 |  |
|  | 6 | 7 | 6 | 1 | 85.71 | 97.62 ±3.39 |
|  |  |  |  |  |  |  |
| Dag1^Flox/-^ ; Sox2^Cre^ | 1 | 3 | 0 | 3 | 0 |  |
|  | 2 | 3 | 0 | 3 | 0 |  |
|  | 3 | 11 | 1 | 10 | 9.09 | 3.03 ±4.80 |
|  |  |  |  |  |  |  |
| Dag1^Bcyto/-^ | 1 | 9 | 9 | 0 | 100 |  |
|  | 2 | 13 | 10 | 3 | 76.92 |  |
|  | 3 | 12 | 11 | 1 | 91.67 | 89.52 ±4.8 |
|  |  |  |  |  |  |  |
| Dag1^Flox/-^ ; Wnt1^Cre^ | 1 | 4 | 4 | 0 | 100 |  |
|  | 2 | 8 | 6 | 2 | 75 |  |
|  | 3 | 11 | 11 | 0 | 100 |  |
|  | 4 | 8 | 7 | 1 | 87.5 |  |
|  | 5 | 9 | 9 | 0 | 100 |  |
|  | 6 | 4 | 4 | 0 | 100 |  |
|  | 7 | 8 | 8 | 0 | 100 |  |
|  | 8 | 7 | 7 | 0 | 100 | 95.31 ±2.94 |
