# Supplementary material for: Dystroglycan is a scaffold for extracellular axon guidance decisions
Source: eLife. 2019 Feb 13;8:e42143. doi: 10.7554/eLife.42143 (PMC6395066; doi:10.7554/eLife.42143)
Supplement: Supplementary file 2. — E12.5 spinal cords were processed for open book preparations and each well-isolated DiI injection site was assessed as showing either normal anterior turning or anterior-posterior randomization. [file elife-42143-supp2.docx]

| Genotype | Embryo # | Injection sites | Normal turn | AP randomization | % normal | Genotype average ±SEM |
| --- | --- | --- | --- | --- | --- | --- |
| *Celsr3^R1548Q/+^* | 1 | 7 | 6 | 1 | 85.7 |  |
|  | 2 | 10 | 8 | 2 | 80 |  |
|  | 3 | 6 | 5 | 1 | 83.33 |  |
|  | 4 | 10 | 9 | 1 | 90 |  |
|  | 5 | 6 | 5 | 1 | 83.33 |  |
|  | 6 | 6 | 5 | 1 | 83.33 |  |
|  | 7 | 4 | 4 | 0 | 100 | 86.5 ±2.52 |
|  |  |  |  |  |  |  |
| *Celsr3^R1548Q/R1548Q^* | 1 | 9 | 2 | 7 | 28.6 |  |
|  | 2 | 10 | 2 | 8 | 25 |  |
|  | 3 | 8 | 3 | 5 | 37.5 |  |
|  | 4 | 7 | 0 | 7 | 0 |  |
|  | 5 | 7 | 3 | 4 | 42.86 |  |
|  | 6 | 7 | 0 | 7 | 0 | 22.32 ±6.35 |

**Table 2: Open book raw data for Celsr3^R1548Q/R1548Q^ mutants**
